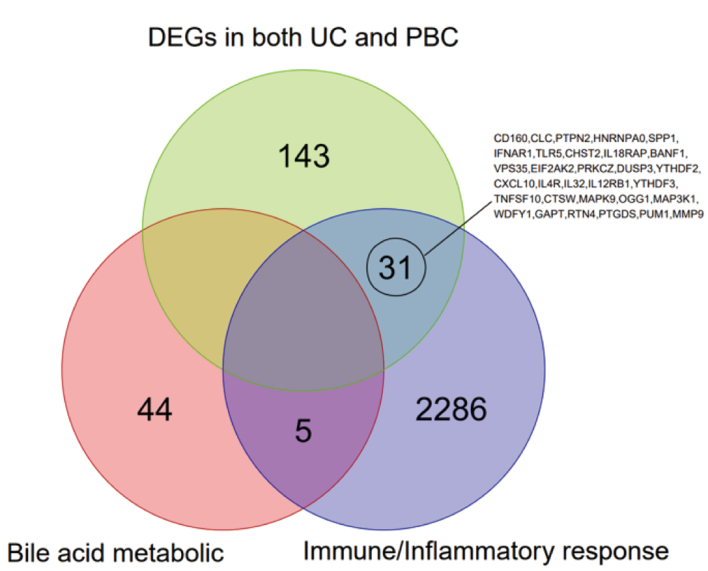# Identification of the underlying molecular mechanisms of primary biliary cholangitis and ulcerative colitis comorbidity

**DOI:** 10.1016/j.gendis.2024.101470

**Published:** 2024-11-26

**Authors:** Weijia Han, Ting Song, Qi Wang, Chunyang Huang

**Affiliations:** a Department of Gastroenterology, Shenzhen Hospital, Southern Medical University, Shenzhen, Guangdong 518000, China; b Beijing Key Laboratory of Emerging Infectious Diseases, Institute of Infectious Diseases, Beijing Ditan Hospital, Capital Medical University, Beijing 100000, China; c Department of Hepatology, The Sixth People's Hospital of Qingdao, Qingdao, Shandong 266033, China

Patients with primary biliary cholangitis (PBC) may have a poor prognosis, with approximately 40% of them developing cirrhosis within 10 years of diagnosis. Recent studies suggest that some metabolites are causally associated with both PBC and ulcerative colitis (UC). UC may increase the risk of PBC in the European population, which may suggest the etiology of PBC.^1^ Meanwhile, poor prognosis in patients with PBC has been shown to be closely associated with UC in many case reports. Additionally, it is hypothesized that the increased levels of lipopolysaccharides and inflammation or immune response are related to the higher permeability of the small intestines in PBC. Moreover, UC is a chronic and progressive inflammatory disease that disrupts the intestinal epithelial barrier and damages the colonic mucosa. Research on the mechanism of comorbid PBC and UC has significant clinical importance for intervention and early recognition of the disease. In this study, we aimed to explore the co-expressed differentially expressed genes (DEGs) and hub genes of PBC and UC, as well as analyze the possible regulatory factors of these genes. This research is conducive to further studying the molecular mechanisms of PBC and UC.

In recent years, with the advancements of bioinformatics, gene chip bioinformatics analysis has become indispensable in the biomedical field. Fresh perspectives on the shared etiology of PBC and UC could be revealed by exploring their common transcriptional patterns. For this investigation, we obtained microarray datasets related to PBC (GSE119600) and UC (GSE169568) from the Gene Expression Omnibus database. By searching the GSE119600 Series Matrix File, which addressed PBC, we identified 968 DEGs, including 418 upregulated and 550 downregulated genes (Fig. S1 and Table S1). By searching the Series Matrix File data file for GSE169568, which addressed UC, we obtained 2637 DEGs, including 1839 upregulated and 798 downregulated genes (Fig. S2 and Table S2). We identified a total of 174 intersecting DEGs, including 52 upregulated and 122 downregulated genes (Fig. S3). Subsequently, the signaling pathways and biological functions of the DEGs involved in disease occurrence and development were analyzed using Gene Ontology and Kyoto Genome Encyclopedia pathway analysis based on the Database for Annotation, Visualization, and Integrated Discovery. The results showed that 112 downregulated DEGs were primarily enriched in pathways, such as cell proliferation, apoptotic, gene expression, and metabolic pathways (Fig. 1A and Table S4); the 52 upregulated DEGs were mainly enriched in inflammatory response, lipopolysaccharide, multicellular development, cytokine–cytokine receptor interaction, and necroptosis pathways (Fig. 1B and Table S4).

**Figure 1:**
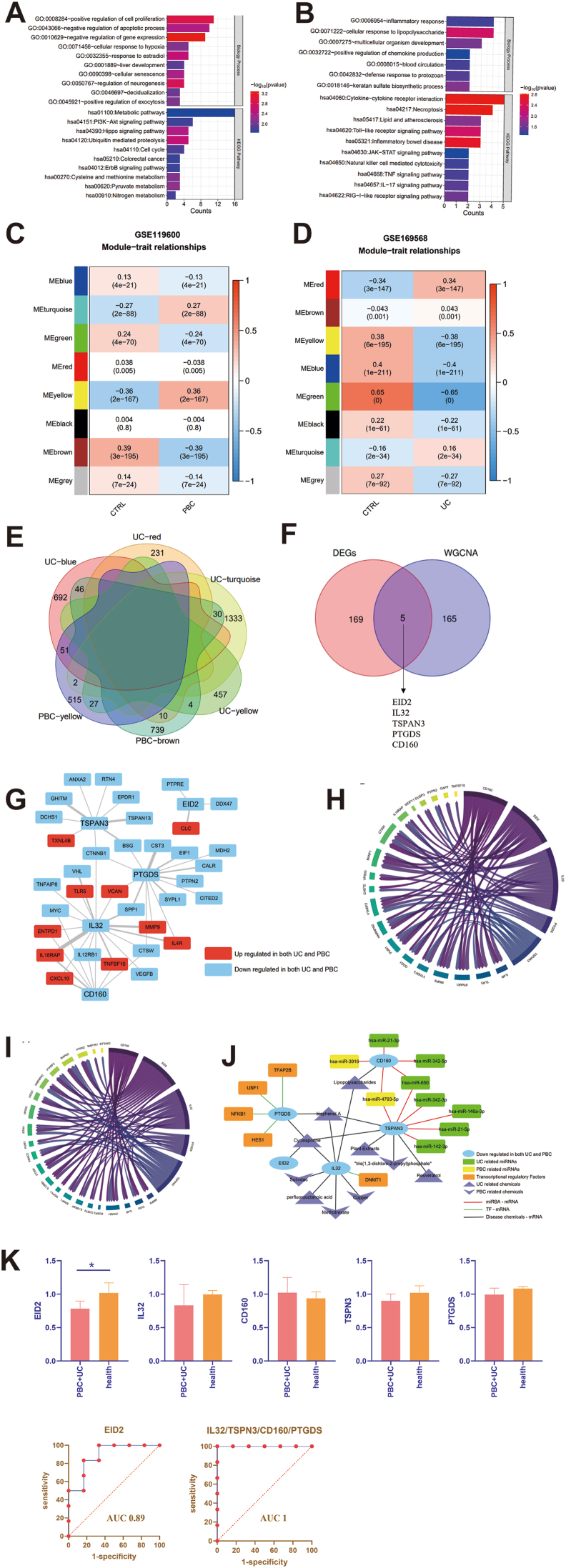
The co-expressed differentially expressed genes (DEGs) and hub genes of primary biliary cholangitis (PBC) and ulcerative colitis (UC). **(A)** Gene Ontology (GO) biological process and Kyoto Genome Encyclopedia (KEGG) signaling pathway enrichment analysis for down-regulated DEGs. The results were deemed statistically significant when the overlap was ≥2 and *P* ≤ 0.05. The horizontal axis represents the number of DEGs involved in the entry. The red color indicates the significant correlation. **(B)** GO biological process and KEGG signaling pathway enrichment analysis for up-regulated DEGs. The results were deemed statistically significant when the overlap was ≥2 and *P* ≤ 0.05. The horizontal axis represents the number of DEGs involved in the entry. The red color indicates the significant correlation. **(C)** WGCNA analysis of PBC. The cutHeight = 0.995. The power was set to 10, and then the gene modules was identified by the tom matrix. **(D)** WGCNA analysis of UC. The cutHeight = 0.995. The power was set to 10, and then the gene modules was identified by the tom matrix. **(E)** Venn diagram of 2 modules of PBC with 4 modules of UC. **(F)** Venn diagram of screened resulting genes of WGCNA compared with DEGs. **(G)** Networks of DEGs interaction. **(H)** Association of DEGs related to immune/inflammatory response and hub genes in PBC. The threshold is *P* ＜ 0.05 and absolute correlation ＞ 0.3. **(I)** Association of DEGs related to immune/inflammatory response and hub genes in UC. The threshold is *P* ＜ 0.05 and absolute correlation ＞ 0.3. **(J)** The transcription factors, mi-RNAs and therapeutic targets of hub genes in PBC and UC. **(K)** The qRT-PCR of hub genes. ∗*P* ＜ 0.05.

We then performed a weighted gene co-expression network analysis (WGCNA) to identify co-expressed gene modules and investigate the relationship between the gene network and the phenotype, as well as identify the core genes in the network. Using the expression profile data of GSE119600, a total of eight gene modules (e.g., yellow) were identified. The gene modules with stronger inverse relationships with PBC were yellow and brown (Fig. 1C and Table S5). Using the expression profile data of GSE169568, a total of eight gene modules (e.g. blue) were discovered. Four gene modules, including blue, green, yellow, and red, had a stronger inverse relationship with UC than the others (Fig. 1D and Table S5). Subsequently, the two gene modules of GSE119600 were intersected with the four modules of GSE169568, and 170 intersecting genes were obtained (Fig. 1E). After comparing the 174 DEGs identified above with these 170 genes, we obtained a total of five down-regulated hub genes (Fig. 1F): EID2, IL32, TSPAN3, PTGDS, and CD160. Next, the interaction between the proteins of the hub genes and the DEGs associated with both diseases was analyzed based on the String datasets. A total of 51 connected pairs were identified. The five hub genes showed high degrees of connection to the 33 identified DEGs, including 10 up-regulated DEGs (e.g., TLR5) and 23 down-regulated DEGs (e.g., MYC) (Fig. 1G).

The pathogenetic mechanisms of PBC involved apoptosis of biliary epithelial cells, immune response caused by pro-inflammatory cytokines, and mitochondrial dysfunction due to oxidative stress caused by toxic bile acids.^2^ Next, a regulatory network was built to identify the DEGs related to immune/inflammatory responses and describe bile acid metabolism using the GSEA database. The results identified 31 DEGs (including the five hub genes) that are related to inflammatory/immune responses, but no DEGs or hub genes were related to bile acid metabolism (Fig. S4). After this, we analyzed the coefficient between the hub genes and the 31 DEGs related to the inflammatory/immune response. Fifty-seven connected pairs were obtained for PBC (Fig. 1H and Table S6) and 58 for UC (Fig. 1I and Table S7). Among these 31 DEGs, IFNAR1, TLR5, and MMP9 had a positive relationship with the hub genes. Just as previously suggested, IFNAR1 expression is potentially beneficial during the early stages of PBC and may enhance barrier destruction in UC. TLR5-related pro-inflammatory cytokines are critical to the breakdown of self-tolerance in PBC, and dysfunction of the TLR5 signaling pathway is related to the alleviation of the symptoms of UC. Furthermore, MMP9 is positively related to hepatic dysfunction in PBC, including connective tissue remodeling and the degradation of collagen, proteoglycans, fibronectin, laminin, and elastin.^3^ Moreover, MMP9 is also involved in injury to the colonic mucosal epithelium in UC.^4^

Furthermore, we predicted miRNAs, transcription factors (TF), and suggested medications or chemicals related to the hub genes. The mRNA-miRNA regulatory network revealed d that the five hub genes had the highest average connectivity with miR-21-3p, miR-342-5p, miR-650, miR-342-3p, miR-146a-3p, miR-21-5p, and miR-142-3p. miR-21 is known to be involved in liver fibrosis and inflammation in PBC, as well as inflammation and apoptosis in UC. The five hub genes had the highest average connectivity with the TF, including TFAP2B, USF1, NFKB1, HES1, and DNMT1. In addition, the five hub genes had the highest average connectivity with the targets, including lipopolysaccharides, cyclosporine, sulindac, and methotrexate (Fig. 1J).

Finally, quantitative real-time PCR (qRT-PCR) was performed in the hub genes. The sequences were produced (Table S8). The results showed a lower level of EID2, IL32, TSPAN3, and PTGDS in patients with both PBC and UC. EID2, as well as the combination of the other four hub genes, had significant sensitivity and specificity as a biomarker for PBC and UC (Fig. 1K). This may provide an important clinical basis for demonstrating the presence of these hub genes as biomarkers in patients with both PBC and UC.

## Funding

This article was supported by the Young and middle-aged Talents Incubation Project 2022 (Youth Innovation) of Beijing Youan Hospital, Capital Medical University, China (No. BJYAYY-YN-2022-09); and the Young and middle-aged Talents Incubation Project 2023 (Youth Innovation) of Beijing You'an Hospital, Capital Medical University, China (No. BJYAYY-YN2023-14), and the WBE Liver Foundation (No. WBE2022018), and the Funding of post-doctor who came to Shenzhen, China (No. UN-KC-2023032).

## CRediT authorship contribution statement

**Weijia Han:** Data curation, Formal analysis, Writing – original draft. **Ting Song:** Data curation. **Qi Wang:** Investigation. **Chunyang Huang:** Writing – review & editing.

## Data availability

The datasets generated during the current study are available from the corresponding author upon reasonable request.

## Conflict of interests

The authors declare no conflict of interests.

## Figures and Tables

**FigS1:**
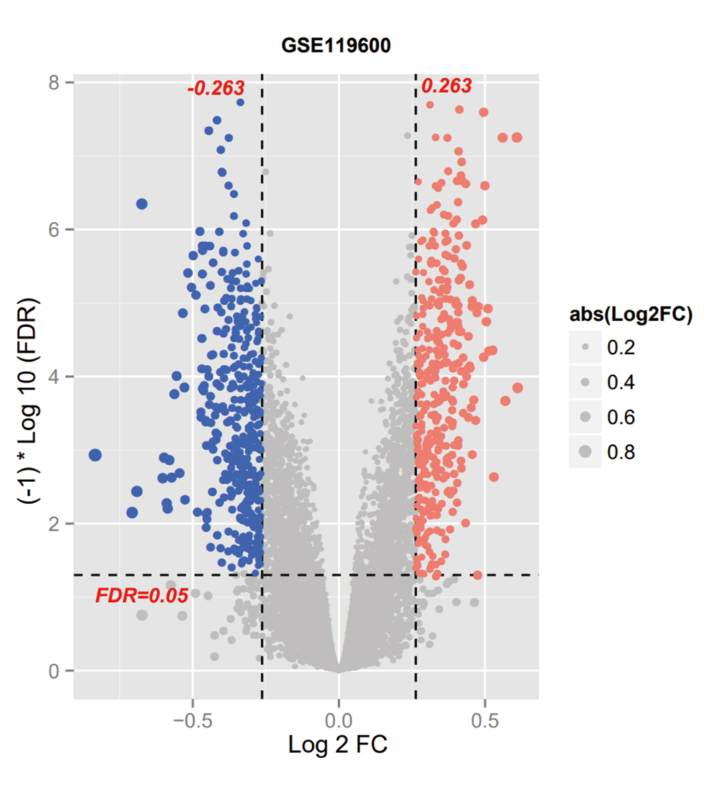


**FigS2:**
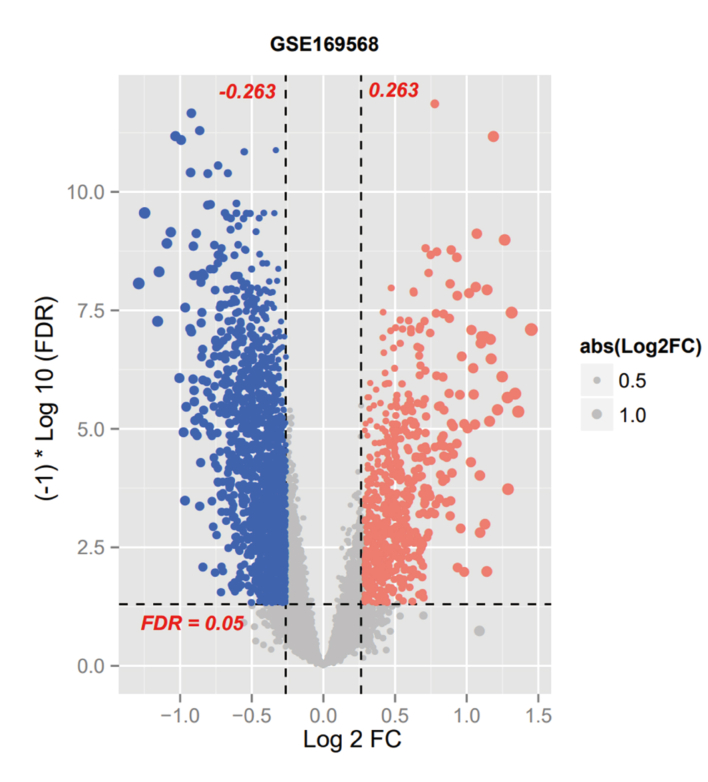


**FigS3:**
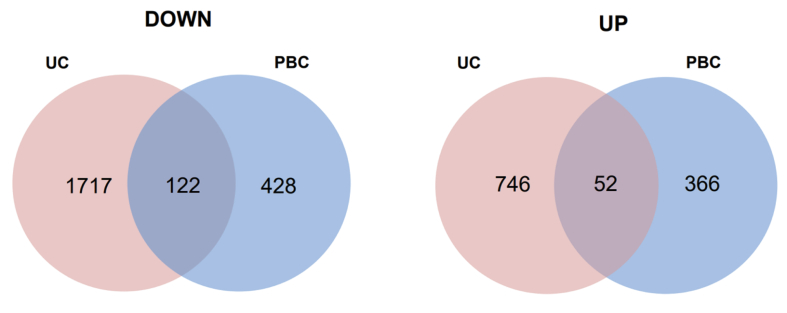


**FigS4:**