# Supplementary material for: Identification of the underlying molecular mechanisms of primary biliary cholangitis and ulcerative colitis comorbidity
Source: Genes Dis. 2024 Nov 26;12(4):101470. doi: 10.1016/j.gendis.2024.101470 (PMC11994317; doi:10.1016/j.gendis.2024.101470)
Supplement: Multimedia component 1 [file mmc1.docx]

**Data download**

The GSE169568 and GSE119600 Series Matrix File data files were downloaded from the GEO public database. GPL10558 is the license for the annotation platform. 90 cases in the PBC group and 58 UC cases were included. The limma package Version 3.34.7 (https://bioconductor.org/packages/release/bioc/html/limma.html） was used to compare the disease affected and normal groups. The screening criteria for DEGs were FDR<0.05 and |logFC|>0.263.

**Analysis of GO and KEGG functions**

Annotation and visualization were performed through the Database for Annotation, Visualization and Integrated Discovery database (DAVID) (<https://david.ncifcrf.gov/>). Then, the signaling pathways and biological functions of the DEGs in disease occurrence and developmentwere analyzed by Gene Ontology (GO) and Kyoto Genome Encyclopedia (KEGG) pathway analysis. The results were deemed statistically significant when the overlap was ≥2 and p≤0.05.

**WGCNA for co-expression network construction**

WGCNA was performed to identify co-expressed gene modules and investigate the relationship between the gene network and phenotype, as well as the core genes in the network. The co-expression network of all genes in the data set was built by the R3.6.1 WGCNA package (<https://cran.r-project.org/web/packages/WGCNA/index.html>) Version 1.61. At least 200 genes with variance for further analysis was identified. The cutHeight=0.995. The power was set to 10, and then the gene modules was identified by the tom matrix.

**Regulatory network analysis of hub gene**

The interaction relationship between the proteins of hub gene, which was the DEGs associated with both disease from limma package and WGCNA package, was carried out by using the STRING (Version:11.0, <http://string-db.org/>). Only the interaction linkage relationships between important DEGs associated with both diseases were retained. The network was visualized by Cytoscape Version 3.9.0 (<http://www.cytoscape.org/> ).

**DEG co-expression network related to immunity/inflammatory response and bile metabolism**

We compared DEGs with the genes related to immune/inflammatory response and BAs metabolism from the MSigDB plate in the GSEA database (<http://software.broadinstitute.org/gsea/downloads.jsp>). Retaining connection was deemed statistically significant p≤0.05 and absolute correlation coefficient＞0.3. The network was visualized by Cytoscape Version 3.9.0 (<http://www.cytoscape.org/> ).

**miRNA,** **TF and potential therapeutic drugs associated with hub genes**

MiRNAs related to UC and PBC were extracted from the HMDD v3.2 database (<http://www.cuilab.cn/hmdd>). The miRWalk 3.0 database (<http://129.206.7.150/> ) was used to predicted the miRNA regulated to hub genes. Transcriptional Regulatory Relationships Unraveled by Sentence-based Text mining (TRRUST) (<https://www.grnpedia.org/trrust/>) database was used to predicted the TF. The Comparative Toxicogenomics Database (TCD) (<http://ctd.mdibl.org/>) database was used to predicted the potential therapeutic drugs. An integrated regulatory network was constructed on miRNA, TF, potential targets and DEGs. The network was then visualized by Cytoscape Version 3.9.0.

**qRT-PCR on hub gene**

The PCR primers of the hub gene were designed. Table 1 showed the sequences. Trizol reagent was used to extract the total cell RNA. SYBR Green PCR was used to reverse transcribe and detect the total RNA. The 2^–ΔΔCt^ method was used to calculate the Ct values obtained by qRT-PCR. GAPDH served as the internal control.

**Figure S1** Volcano map of DEGs in PBC, blue indicates down-regulated DEGs, and red indicates up-regulated DEGs.

**Figure S2** Volcano map of DEGs in UC, blue indicates down-regulated DEGs, and red indicates up-regulated DEGs.

**Figure S3** Venn diagram identifies co-upregulated and co-downregulated DEGs. The screening criteria for DEGs were FDR < 0.05 and |logFC| > 0.263.

**Figure S4** DEG related to immune/inflammatory response and bile acids metabolism.
